# High carbohydrate and high fat diets protect the heart against ischaemia/reperfusion injury

**DOI:** 10.1186/s12933-014-0109-8

**Published:** 2014-07-18

**Authors:** Ruduwaan Salie, Barbara Huisamen, Amanda Lochner

**Affiliations:** 1 Department of Biomedical Sciences (Section: Medical Physiology), Faculty of Medicine and Health Sciences, University of Stellenbosch, Tygerberg 7505, South Africa

**Keywords:** Obesity, Insulin resistance, Cardioprotection, Infarct size, Diet-induced obesity, High fat diet, RISK pathway

## Abstract

**Background:**

Although obesity is still considered a risk factor in the development of cardiovascular disorders, recent studies suggested that it may also be associated with reduced morbidity and mortality, the so-called “obesity paradox”. Experimental data on the impact of diabetes, obesity and insulin resistance on myocardial ischaemia/reperfusion injury are controversial. Similar conflicting data have been reported regarding the effects of ischaemic preconditioning on ischaemia/reperfusion injury in hearts from such animals. The aim of the present study was to evaluate the susceptibility to myocardial ischaemia/reperfusion damage in two models of diet-induced obesity as well as the effect of ischaemic and pharmacological preconditioning on such hearts.

**Methods:**

Three groups of rats were fed with: (i) normal rat chow (controls) (ii) a sucrose-supplemented diet (DIO) (iii) a high fat diet (HFD). After 16 weeks, rats were sacrificed and isolated hearts perfused in the working mode and subjected to 35 min regional ischaemia/60 min reperfusion. Endpoints were infarct size and functional recovery. Infarct size was determined, using tetrazolium staining. Activation of PKB/Akt and ERKp44/p42 (RISK pathway) during early reperfusion was determined using Western blot. Statistical evaluation was done using ANOVA and the Bonferroni correction.

**Results:**

Infarct sizes of non-preconditioned hearts from the two obese groups were significantly smaller than those of the age-matched controls. Ischaemic as well as pharmacological (beta-adrenergic) preconditioning with a beta2-adrenergic receptor agonist, formoterol, caused a significant reduction in infarct size of the controls, but were without effect on infarct size of hearts from the obese groups. However, ischaemic as well as beta-preconditioning caused an improvement in functional performance during reperfusion in all three groups. A clear-cut correlation between the reduction in infarct size and activation of ERKp44/p42 and PKB/Akt was not observed: The reduction in infarct size observed in the non-preconditioned hearts from the obese groups was not associated with activation of the RISK pathway. However, beta-adrenergic preconditioning caused a significant activation of ERKp44/p42, but not PKB/Akt, in all three groups.

**Conclusions:**

Relatively long-term administration of the two obesity-inducing diets resulted in cardioprotection against ischaemia/reperfusion damage. Further protection by preconditioning was, however, without effect on infarct size, while an improvement in functional recovery was observed.

## Background

Obesity is considered to be a serious risk factor in the development of cardiovascular disorders and is traditionally regarded to impact negatively on the outcome of myocardial ischaemia. However, despite the evidence for a higher prevalence of obesity in myocardial ischaemia patients, a number of recent publications suggested that obesity in humans with ischaemic heart disease is associated with reduced morbidity and mortality, the so-called “obesity paradox” [[1]-[3]]. The mechanism underlying this paradox is complex and remains unclear.

Experimental data on the impact of diabetes, obesity and insulin resistance on myocardial ischaemia/ reperfusion injury (IRI) are also controversial (for reviews see references [[4],[5]]). In contrast to the vast amount of research done to assess the susceptibility of the heart to ischaemia/reperfusion damage in types I and II diabetes [[4]-[6]], relatively little is known about the effects of obesity per se in this scenario. Experimental studies reported a variety of outcomes. For example, in both types I and II diabetes, hearts have been reported to be more [[7]-[10]] or less [[11]-[15]] susceptible to ischaemic damage or not different from normoglycaemic controls [[12],[16],[17]]. In the case of experimental obesity and insulin resistance similar discrepancies have been reported. A decreased myocardial tolerance to ischaemia-reperfusion damage was observed in *in vivo* [[18],[19]] and *ex vivo* [[20]-[22]] studies using hyperphagia-induced obese insulin resistant male rats. Similar findings were reported in other animal models [[9],[23]-[26]]. Collectively, these studies have shown that an increased infarct size is associated with concomitant poor functional recovery in hearts from obese animals compared to the controls. In contrast to the above, smaller infarcts and improved functional recovery during reperfusion were reported by Donner and coworkers [[27]], using a hyperphagia-induced obese rat model. A possible explanation for the variation in susceptibility to ischaemia/reperfusion damage may be differences in the age of the animals and the duration of obesity.

Similar conflicting data were obtained when the possibility of protection of the diabetic heart by ischaemic preconditioning (IPC) was investigated. A few studies reported preconditioning-mediated protection [[28]-[32]] in hearts from streptozotocin-induced diabetic rats. However, a considerable number of studies could not demonstrate this phenomenon, for example, IPC failed to exert protective effects in Zucker obese [[13],[26]], streptozotocin-induced diabetic rats [[15]] and dogs [[8]]. Using Goto-Kakizaki rats, Tsang and coworkers [[14]] reported that diabetic rats could be preconditioned but required more ischaemia/reperfusion cycles than controls to induce protection. Interestingly, failure of myocardial protection by IPC of streptozotocin-induced diabetic hearts, was attributed to rapid and extensive loss of ferritin during sustained ischaemia after a preceding preconditioning protocol [[33]].

Despite the plethora of reports on the ability to precondition hearts from diabetic animals [[28]], as far as we know no information is available regarding preconditioning-induced cardioprotection in hyperphagia-induced obesity.

It is now generally accepted that the reduction in injury during reperfusion is characterized by activation of the reperfusion injury salvage kinase pathway (RISK) [[34],[35]]. Central to this pathway is activation of the prosurvival/anti-apoptotic kinase PKB/Akt, which is also a key enzyme in the insulin signalling pathway [[36]]. However, conclusions about the involvement of the RISK pathway in the response to IPC is hampered by the fact that not only the models of diabetes differed, but tissue samples were taken at different times during the experimental protocol. For example, samples were collected at the end of the stabilization time or after the IPC stimulus [[14]], during reperfusion after sustained ischaemia [[37]] or under baseline conditions [[27],[38]]. Baseline activation of ERK was found to be increased in streptozotocin-induced diabetes [[38]] while both ERKp44/p42 and PKB/Akt phosphorylation were reported to be lower in rats on a high fat diet [[19]]. ERK activation during reperfusion of streptozotocin-induced diabetic hearts is dependent on the duration of hyperglycaemia: an increment is seen after 4 weeks followed by a significant reduction after 20 weeks [[37]], while PKB/Akt activation showed a similar early stimulation followed by a reduction [[12]].

In view of the above-mentioned controversies and caveats in our current knowledge, the aim of the present study was to evaluate the susceptibility to ischaemia/reperfusion damage in two models of diet-induced obesity as well as the effect of ischaemic and pharmacological preconditioning. The most significant observations were that rats on obesity-inducing diets were characterized by protection against myocardial ischaemia/reperfusion and insensitivity to the effects of prior ischaemic or pharmacological preconditioning.

## Materials and methods

### Animals

Age and weight matched male Wistar rats were used in this study. This study was approved by the Committee for Ethical Animal Research of the Faculty of Health Sciences, University of Stellenbosch. The animals were obtained from the University of Stellenbosch Central Research Facility. They received water and food ad libitum (light/dark cycle 6 h00-18 h00; temperature 22°C; humidity 40%). Animals were treated according to the revised South African National Standard for the Care and Use of Animals for Scientific Purposes (South African Bureau of Standards, SANS 10386, 2008). Rats weighing 200 ± 20 g were divided into three groups: (i) control rats received a standard commercial rat chow, (ii) diet-induced obese rats received a sucrose-supplemented diet (DIO), (iii) high fat diet rats received the DIO diet supplemented with Holsum cooking fat (HFD). Holsum cooking fat contained 68% saturated fat, 26% mono-unsaturated fat and 5% poly-unsaturated fat. The animals were fed their respective diets for 16 weeks. The DIO diet was prepared by addition of condensed milk and sucrose to the standard rat chow as described by Pickavance and coworkers [[39]], while the HFD was obtained by adding Holsum cooking fat to the DIO diet. The composition of the diets is summarized in Table 1.

**Table 1:** Composition of diets

	**Control**	**DI0**	**HFD**
Total fat (g/100 g)	4.8	4.6	11.5
Saturated fat (g/100 g)	0.9	2.8	7.6
Cholesterol (mg/100 g)	2.2	10	13
% protein	17.1	9.4	8.3
% carbohydrate	34.6	46	42
Sucrose (g/100 g)	5.3	23.3	20.4

Control: normal rat chow.

DI0: rat chow + sucrose.

HFD: rat chow + sucrose + cooking fat.

Analyses were done commercially (Microchem, Cape Town).

### Chemicals

Routine chemicals (pro analysi) were obtained from Merck Chemical Co (Cape Town, RSA). Antibodies (ERKp44/p42, phospho-ERKp44/p42(Thr-202/Tyr-204), PKB/Akt and phospho-PKB/Akt (Ser-473) were purchased from Cell Signaling Technology, Beverly, MA, USA). Horse-radish peroxidase labeled secondary antibody, ECL and hyperfilm were from Amersham, Life Science.

### Experimental procedure

After 16 weeks of feeding, non-fasted rats were weighed and anaesthetized with pentobarbital (30 mg/rat) and the blood glucose measured using the tail prick method and a glucometer, as described below. They were then sacrificed, the blood collected and the hearts removed for subsequent perfusion in the working mode. The visceral fat was collected and weighed. In a separate series of experiments rats were fasted overnight before sacrifice. The blood was collected, centrifuged, the serum collected and stored at -80°C for subsequent biochemical analyses. For evaluation of the baseline RISK pathway, hearts were freeze-clamped immediately after removal and stored at −80°C until analysis.

### Perfusion technique

Hearts were perfused as described before [[40]]. Briefly, a modified Krebs-Henseleit bicarbonate buffer was used, containing (in mM): NaCl 119; NaHCO_3_ 24.9; KCl 4.7; KH_2_PO_4_ 1.2; MgSO_4_.7H_2_O 0.59; Na_2_SO_4_ 0.59; CaCl_2_ 1.25; glucose 10. The buffer was oxygenated with a 95%oxygen/5%CO_2_ gas mixture at 37°C.

All perfused hearts were stabilized by retrograde perfusion for 15 min, followed by perfusion for 15 min in the working mode, during which time the aortic and coronary flow rates were measured. Thereafter hearts were perfused for either 20 min retrogradely (non-preconditioned (NPC), or for 10 min retrogradely, followed by 5 min global ischaemia/5 min reperfusion (IPC) or for 10 min retrogradely, followed by 5 min formoterol (1nM), 5 min washout (beta-preconditioning, BPC). After preconditioning, hearts were perfused for an additional 10 min in the working mode to allow measurement of function before the onset of ischaemia. Hearts were then subjected to 35 min regional ischaemia (36.5°C) and 60 min reperfusion for measurements of functional recovery (at 20 and 30 min reperfusion) and infarct size. Systolic pressure and heart rate were measured through a side-arm of the aortic cannula connected to a Viggo-Spectramed pressure transducer coupled to a computer system.

For evaluation of ERKp44/p42 and PKB/Akt activation, hearts were stabilized as described above, but subjected to 15 min global ischaemia, followed by 10 min reperfusion and then freeze-clamped for subsequent Western blotting.

### Determination of infarct size

Myocardial infarct size was determined as described previously, using tetrazolium staining [[41]]. Each heart was cut in 2 mm thick slices. For each slice the left ventricle area at risk and the infarcted areas were determined using computerized planimetry (UTHCSA Image Tool programme, University of Texas Health Science Center at San Antonio, TX, USA).

### Western blot analyses

Hearts from each group were collected either after sacrifice (baseline) or after 10 min reperfusion following 15 min global ischaemia. A so-called negative control, prepared from an untreated control heart perfused in the Langendorff mode for 15 min, was included in each blot. Cytosolic PKB/Akt and ERKp44/p42 were extracted from frozen left ventricular tissue by pulverization and homogenization in 600–900 μl lysis buffer using a Polytron homogenizer as described before [[42]]. Samples were centrifuged at 1000 g for 10 min to obtain the supernatant which was used for Western blotting. The protein content was determined using the Bradford technique [[43]]. The lysates were diluted in Laemmli sample buffer. Western blotting was done as described before [[42]]. Membranes were routinely stained with Ponceau red for visualization of proteins and to confirm adequate transfer and equal loading. Films were densitometrically analyzed (UN-SCAN-IT™, Silk Scientific Inc, Orem, Utah, USA). Protein activation was expressed as a ratio of phospho- to total protein.

For evaluation of activation of ERKp44/p42 and PKB/Akt during reperfusion, 4–5 hearts were studied in each series. For comparison purposes, each blot included the same control and the data obtained from all experimental groups were expressed as a ratio of the control heart.

### Biochemical analyses

Fasting blood glucose was measured using a glucometer (Gluco PlusTM, distributed by CIPLA DIBCARE, Bellville, South Africa). Serum insulin levels were determined using a competitive radioimmunoassay (RIA) (Coat-A-Count® Insulin, Siemens Healthcare Diagnostic Products Inc., LA, USA) according to the manufacturer’s instructions. Fasting serum triglycerides were determined using CardioCheck_ PA analyser (PolymerTechnology City, Indianapolis, IN, USA). For this test, 40 μL of fresh blood was collected and placed on a lipid test strip (PTS PanelsTM; Polymer Technology City), for measurement of triglycerides.

### Statistical analysis

Results were processed using GraphPad Prism statistical software (versions 5 and 6). All results were expressed as mean ± standard error of the mean (SEM). For comparisons, an analysis of variance (ANOVA), followed by the Bonferroni correction was applied. A p-value of p < 0.05 was considered significant.

## Results

### Biometric parameters

Both diets, when given for a period of 16 weeks, caused significant increases in body weight when compared to rats receiving normal rat chow (DIO: 12% and HFD: 21%). This was also associated with significant increases in visceral fat in both groups. Fasting blood glucose levels were slightly, but significantly higher in the obese groups, whereas serum triglycerides and insulin levels were also significantly higher in the DIO and HFD rats respectively (see Table 2).

**Table 2:** Effects of diets on body weight and blood parameters

	**Non-diet controls (NDC)**	**Diet-induced obese rats (DIO)**	**HF diet rats (HFD)**
Body wt (g) (16)	418 ± 10	470 ± 18*	507 ± 16*
Visceral fat (g) (16)	15.9 ± 1.2	23.9 ± 1.7**	37.4 ± 2.7**^†^
Blood glucose (mmol/L)(16)	5.29 ± 0.1	6.37 ± 0.4*	6.38 ± 0.18**
Serum triglyceride (mmol/L) (10)	0.35 ± 0.04	1.04 ± 0.17*	0.77 ± 0.07*
Serum insulin (μIU/ml) (16)	22.02 ± 3.03	58.6 ± 6.4*	37.08 ± 2.83 83*

Number in brackets indicates number of animals. Animals were fasted overnight.

*p < 0.05 vs NDC.

**p < 0.001 vs NDC.

^†^p < 0.001 vs DI0.

### Effect of diet on infarct size and functional recovery

#### Infarct size

**Figure 1:**
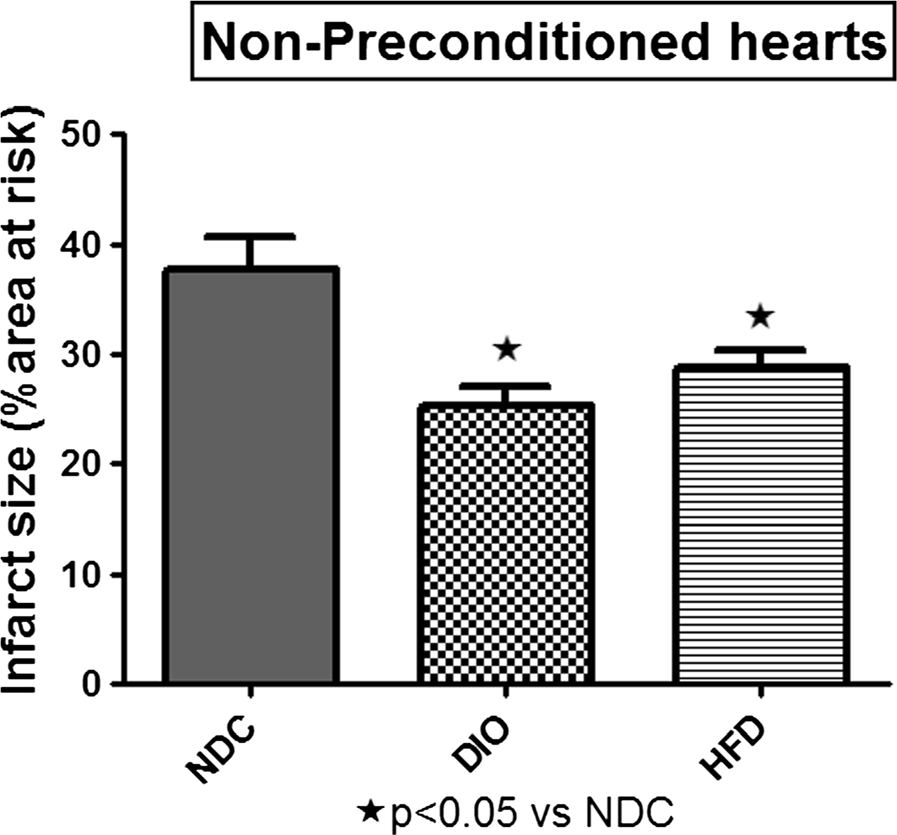
**Effect of diet on infarct size of non-preconditioned (NPC) hearts after 35 min regional ischaemia/60 min reperfusion.** Abbreviations: NDC: non-diet controls; DIO: diet-induced obesity: HFD: high fat diet.

**Figure 2:**
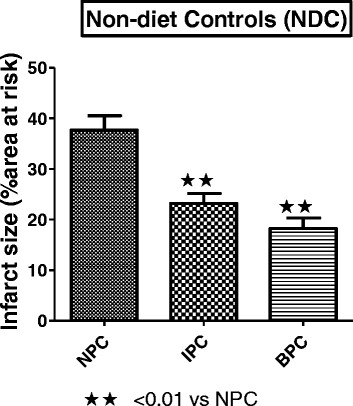
**Effect of ischaemic and beta-preconditioning on infarct size of NDC after 35 min regional ischaemia/.60 min reperfusion.** Abbreviations: NPC: non-preconditioned; IPC: ischaemic preconditioning; BPC: beta-preconditioning.

The percentage of the area at risk did not differ between the nine groups and averaged 50.8 ± 0.8. Comparison of NPC hearts from the control and the two obese groups, showed that the infarct sizes of DIO and HFD hearts after exposure to 35 min regional ischaemia, were significantly smaller than those from rats on a control diet (NDC). (NDC: 37.69 ± 2.86; DIO 25.31 ± 1.75; HFD 28.81 ± 1.58, p < 0.05 vs NDC) (Figure 1). IPC as well as BPC caused a significant lowering in infarct size of hearts from non-diet controls (NDC) (p < 0.01 respectively) (Figure 2), when compared to those of NPC hearts.

**Figure 3:**
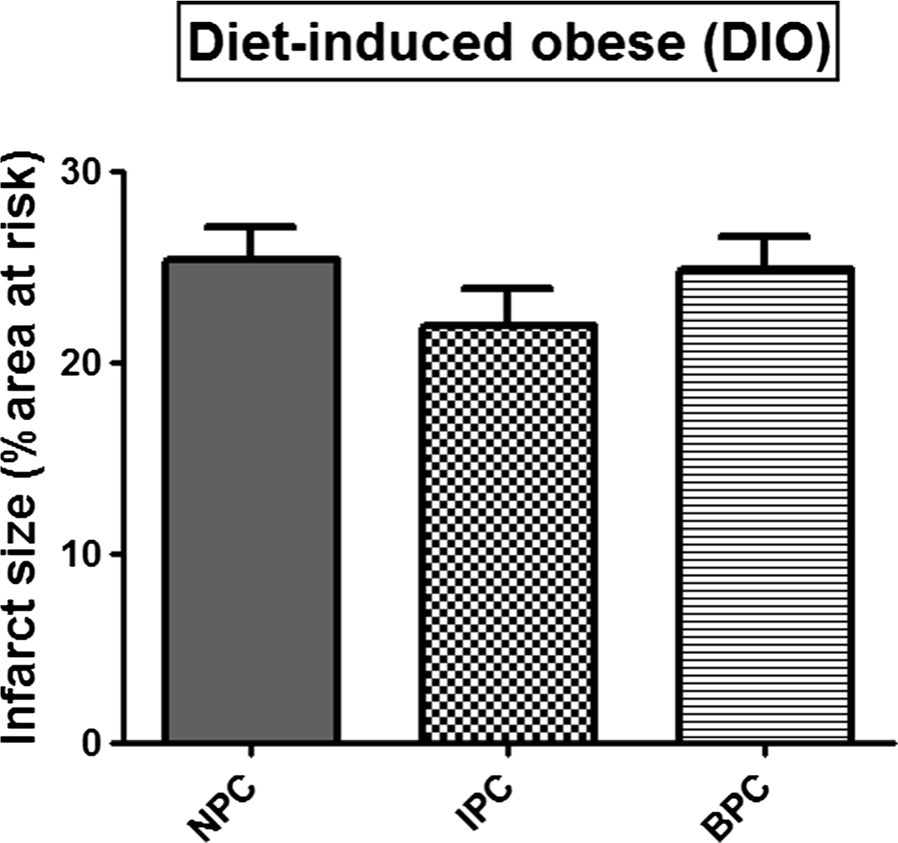
**Effect of IPC and BPC on infarct size of DIO hearts after 35 min regional ischaemia/60 min reperfusion.** Abbreviations: see Figures 1 and 2.

**Figure 4:**
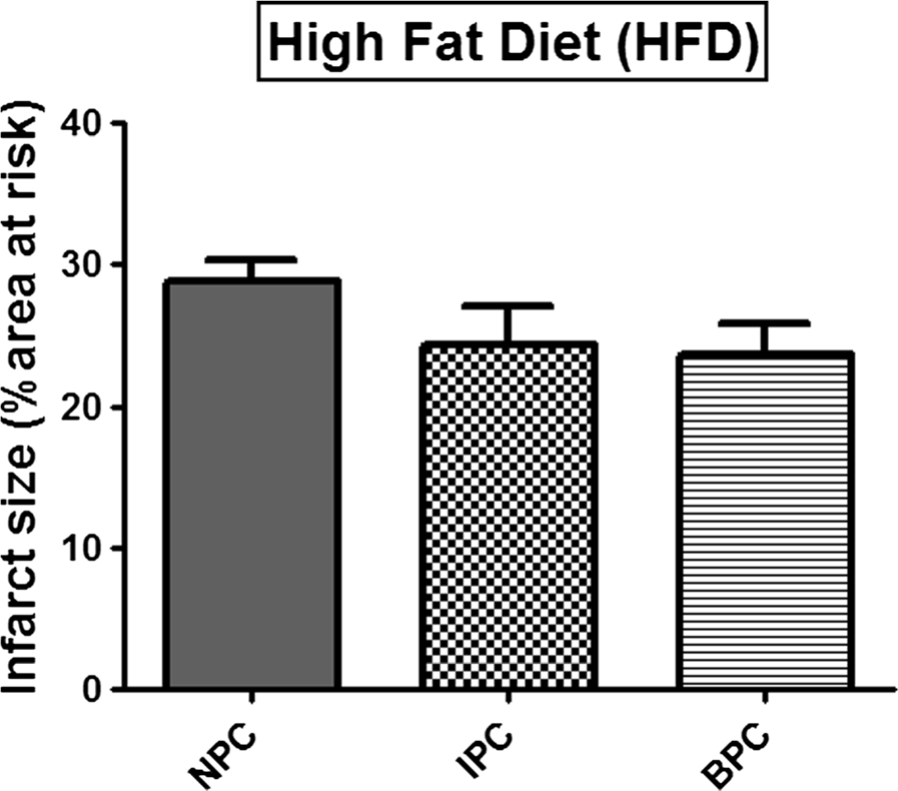
**Effect of IPC and BPC on infarct size of HFD hearts after 35 min regional**.

However, hearts from rats on the DIO and HFD diets did not respond to the preconditioning protocols: with both IPC and BPC infarct sizes were similar to those of their respective NPC groups (Figures 3 and 4).

#### Functional recovery

Parameters of cardiac function during the control stabilization period were evaluated at two time points: (i) before the preconditioning protocol and (ii) before the onset of sustained ischaemia. Since these values did not differ significantly in the NDC, DIO and HFD groups, the values obtained at the first time point were included in Table 3. The parameters of cardiac performance during this period were similar in the NDC, DIO and HFD groups and were therefore pooled. Exposure to 35 min regional ischaemia caused a significant reduction in coronary flow, aortic flow, cardiac output and total work during reperfusion of NPC hearts from all three groups when compared to values obtained during the control period.

**Table 3:** Effects of diets and preconditioning on myocardial function before and after ischaema

	**CF**	**AO**	**CO**	**HR**	**PSP**	**TW**	**Number of hearts producing AO during reperfusion**
	**(ml/min)**	**(ml/min)**	**(ml/min)**	**(beats/min)**	**(mmHg)**	**(mmHg)**	
**Before ischaemia** (45)	20.5 ± 0.5	37.6 ± 1.0	58.2 ± 1.1	257 ± 4	92 ± 1	11.6 ± 0.39	
**After ischaemia**							
NDC NPC	9.1 ± 4.1*	4.8 ± 2.9***	13.9 ± 6.8*	167 ± 68	49 ± 20	3.09 ± 1.31*	2/5
IPC	15.2 ± 0.8	23.2 ± 2.3*^†^	38.4 ± 3.0*^†^	301 ± 34	83 ± 2	7.49 ± 0.86^†^	5/5
BPC	16.3 ± 1.0	16.8 ± 4.7**^†^	33.5 ± 6.0*	252 ± 18	85 ± 3	6.58 ± 1.35	5/5
DI0 NPC	6.4 ± 4.1**	4.0 ± 2.5***	10.4 ± 6.6*	96 ± 59	32 ± 20*	1.88 ± 1.21***	3/5
IPC	15.2 ± 4.1	20.0 ± 5.2**^†^	35.2 ± 1.1*^†^	211 ± 53	71 ± 18	7.07 ± 1.78^†^	4/5
BPC	15.8 ± 1.2	15.2 ± 2.3**^†^	31 ± 3.3*^†^	278 ± 13	85 ± 2	5.75 ± 0.81^†^	5/5
HFD NPC	8.8 ± 3.7*	4.8 ± 2.3***	14.4 ± 6.0**	141 ± 58	49 ± 20	2.66 ± 1.09***	2/5
IPC	13.6 ± 3.5	13.2 ± 5.2***	26.8 ± 7.8*	210 ± 55	69 ± 17	5.18 ± 1.50*	4/5
BPC	8.7 ± 3.9*	9.0 ± 5.4***	17.0 ± 8.4**	126 ± 57*	42 ± 19	3.36 ± 1.64**	3/6

Abbreviations: *CF* Coronary flow, *AO* Aortic output, *CO* Cardiac output, *HR* heart rate, *PSP* peak systolic pressure, *TW* total work.

*NPC* non-preconditioned, *IPC* ischaemic preconditioning, *BPC* beta-preconditioning.

Five-six hearts were studied in each series. Values obtained before ischaemia were grouped together.

*p < 0.05 vs Before; **p < 0.01 vs Before; ***p < 0.001 vs Before; ^†^p < 0.05 vs NPC.

Despite the marked differences in the infarct sizes of NPC hearts (smaller infarcts in DIO and HFD hearts), no significant differences were observed when the post-ischaemic functional parameters of these groups were compared.

However, IPC did improve the mechanical performance of the NDC and DIO groups as evidenced by the significant increases in aortic flow, cardiac output and total work performance during reperfusion, when compared to their respective NPC counterparts. These parameters also increased during reperfusion of IPC HFD hearts, but the changes were not significantly different from those of the corresponding NPC hearts.

In the case of beta-adrenergic preconditioning hearts from the DIO group showed significant improvement in aortic flow, cardiac output and total work during reperfusion, while the aortic output only was significantly higher in the NDC group, when compared to their corresponding NPC hearts. The improvement in the HFD hearts was not significant.

### Activation of the RISK pathway during reperfusion

For evaluation of the RISK pathway during reperfusion, preference was given to a model of global (as opposed to regional) ischaemia, due to the larger amounts of damaged tissue available. Using this model, we previously showed a good correlation between infarct size and activation of this pathway see ref [[42]]. Thus hearts were freeze-clamped at 10 min reperfusion after exposure to 15 min global ischaemia. Previous studies from our laboratory showed maximal activation of the kinases at this time point.

**Figure 5:**
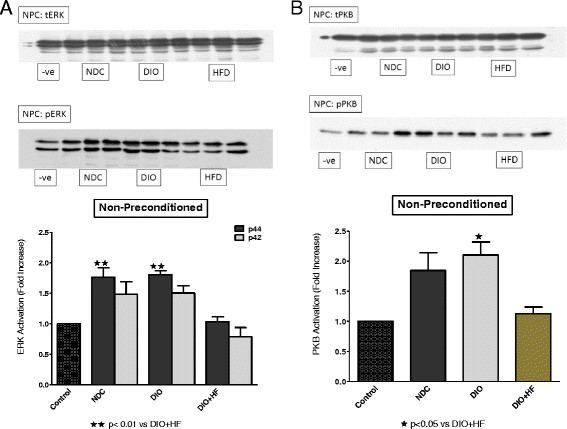
**(A) ERKp44/p42 and (B) PKB activation in non-preconditioned NDC, DIO and HFD hearts.** Abbreviations: see Figures 1 and 2; −ve: negative control. tERK: total ERK; pERK: phospho-ERK. Representative blots are shown; n = 4-5/group.

**Figure 6:**
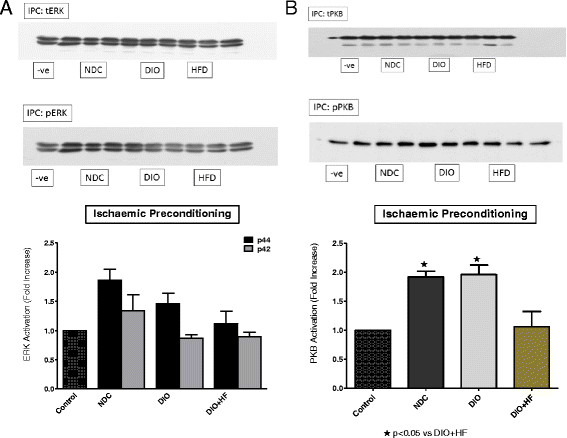
**(A) ERKp44/p42 and (B) PKB activation in ischaemic preconditioned NDC, DIO and HFD hearts.** Abbreviations: see Figures 1, 2 and 5. Representative blots are shown; n = 4-5/group.

**Figure 7:**
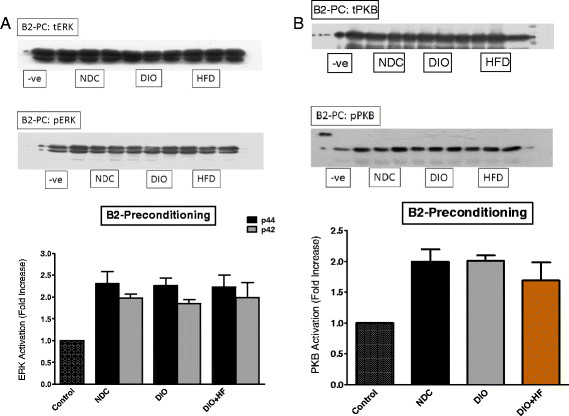
**(A) ERKp44/p42 and (B) PKB activation in beta-adrenergic preconditioned NDC, DIO and HFD hearts.** Abbreviations: see Figures 1, 2 and 5.

**Figure 8:**
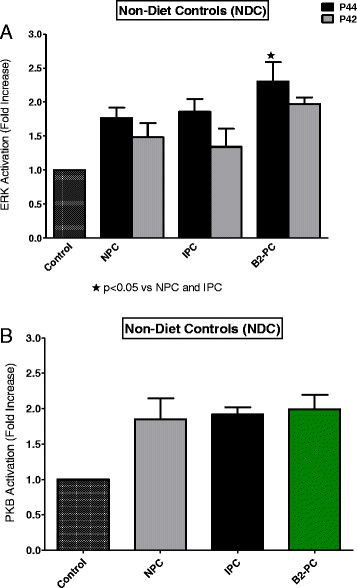
**Non-diet controls: effect of NPC, IPC and BPC on ERKp44/p42 (A) and PKB (B) activation.** Abbreviations: see Figures 1, 2 and 5.

**Figure 9:**
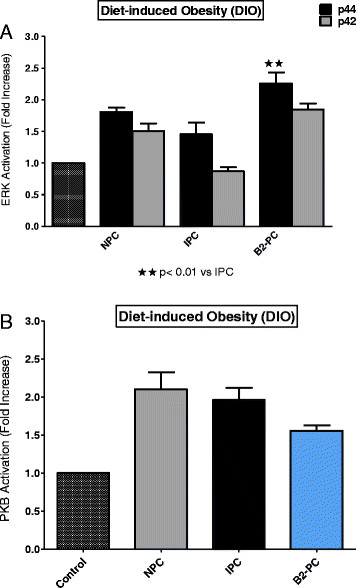
**DIO: effect of NPC, IPC and BPC on ERKp44/p42 (A) and PKB (B) activation.** Abbreviations: see Figures 1, 2 and 5.

**Figure 10:**
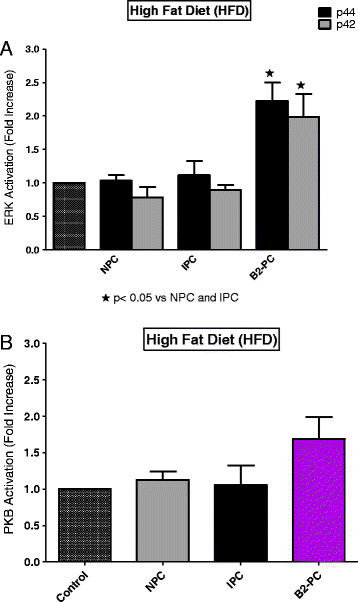
**HFD: effect of NPC, IPC and BPC on ERKp44/p42 (A) and PKB (B) activation.** Abbreviations: see Figures 1, 2 and 5.

Comparisons between the three groups were done as follows: (i) the activation patterns of ERKp44/p42 and PKB/Akt in NPC, IPC and BPC hearts were compared separately in the NDC, DIO and HFD groups (Figures 5, 6 and 7) (ii) the effect of IPC and BPC on the activation of the kinases in each group was compared with those of the corresponding NPC hearts (Figures 8, 9 and 10). For this purpose values obtained in the blots shown in Figures 5, 6 and 7 were used. For all blots, the activation pattern of a control perfused heart was used for normalization of the data to allow comparison of values obtained from different blots.

The expression and activation of ERKp44/p42 and PKB/Akt at baseline did not differ between the three groups studied (results not shown). Activation of both ERKp44/p42 and PKB/Akt during reperfusion was observed in non-preconditioned NDC and DIO hearts, but not in the HFD hearts (Figure 5A,B). Ischaemic preconditioning elicited a significant activation of PKB/Akt, but not ERKp44/p42, in NDC and DIO hearts. No activation of either kinase was visible in the HFD hearts (Figure 6A,B). These data indicated that the activation pattern of the RISK pathway was very similar in hearts from the NDC and DIO groups, with the HFD group showing no activation of these kinases when reperfused.

However, in the case of beta-adrenergic preconditioning, activation of ERKp44/p42 and PKB/Akt was seen in all three groups, but no significant differences were observed between the groups (Figure 7A,B).

Comparison of the activation pattern of the RISK pathway in NPC hearts with those of preconditioned hearts from each group is shown in Figures 8, 9 and 10. An IPC protocol had no effect on the activation pattern of either kinase in all three groups, compared to the NPC groups. BPC by formoterol caused significant increases in ERKp44, but not PKB/Akt phosphorylation (Figures 8, 9 and 10) in all three groups.

## Discussion

The hyperphagia-induced model of obesity has been used by several workers in the past [[39]], including ourselves [[18],[20]] and is characterized by moderate weight gain, increased serum insulin, triglyceride (see Table 2) and free fatty acid (data not shown) levels. Contrary to our expectations, addition of the cooking fat Holsum to the DIO diet did not cause significant changes in the biometric parameters when compared to the DIO diet alone. Both diets caused a small but significant increase in blood glucose levels, although these animals were not diabetic. Thus the rats show some of the characteristics of the metabolic syndrome being obese, insulin resistant and hyperlipidaemic, but not diabetic.

A number of interesting observations were made: (i) relatively long-term administration of the two obesity-inducing diets had no effect on the baseline mechanical performance of isolated working hearts, when compared with rats fed a control diet (NDC); (ii) the infarct sizes of NPC hearts subjected to 35 min regional ischaemia, were significantly lower in the DIO and HFD groups than in hearts from rats receiving normal rat chow, suggesting diet-induced cardioprotection; (iii) in contrast with NDC, hearts from the DIO and HFD groups could not be further protected against I/R injury by either prior IPC or BPC, using infarct size as endpoint. The latter two interventions however caused an improvement in the functional recovery during reperfusion in the NDC and DIO groups, but not in the HFD group.

### Effect of obesity on baseline contractile function

In contrast to the reported reduction in baseline mechanical function in rats receiving a similar high carbohydrate diet (DIO) for the same time period (16 weeks) [[20],[44]], in the present study, hearts from both the DIO and HFD groups exhibited a mechanical performance similar to those of the NDC under these conditions (Table 3). However when administering the same DIO diet for a period of 32 weeks, Du Toit and coworkers [[27]] could no longer detect the contractile dysfunction and insulin insensitivity as mentioned above [[20],[44]], despite significant increases in body weight and visceral adiposity. Similarly, du Toit and coworkers reported that a high fat obesogenic diet for 32 weeks had no deleterious effects on basal myocardial function, whether evaluated *in vivo* or *in vitro* [[19]]. The reasons for these observations are not clear.

Indications are that changes in myocardial metabolic patterns may underlie some of the discrepancies reported. For example, the mechanical dysfunction reported in rats fed a Western diet with high fat and reduced carbohydrates [[45]], was attributed to impaired fatty acid oxidation. Indeed, decreased long chain fatty acid oxidation during reperfusion has been shown to impair postischaemic recovery in sucrose-diet insulin resistant hearts [[46]]. On the other hand, others have reported that hearts from obese insulin resistant mice showed well-preserved function when perfused with palmitate plus insulin [[47],[48]]. Our results, showing no difference between the baseline function of the NDC and obese groups, obtained with glucose as the only substrate *ex vivo*, were surprising in view of the serum lipid abnormalities present *in vivo* in both diet groups after 16 weeks (Table 2), which may have predisposed hearts of obese rats towards fatty acid metabolism. Preliminary studies, using palmitate (1.2 mM/3%albumin) as substrate, yielded similar results viz a smaller infarct size and inability to precondition hearts from obese rats [Lochner A, Genade S: Unpublished observations].

### Response to ischaemia/reperfusion

The results obtained suggest that hearts from both obese groups have developed tolerance against ischaemic damage over a sixteen week period, as demonstrated by infarct sizes of NPC hearts being significantly lower than those of hearts from NDC animals (see Figures 1, 2, 3 and 4). The possible existence of a so-called “metabolic preconditioning” has recently been discussed in a review by Balakumar [[28]] and is suggested to occur in the acutely diabetic myocardium, while the chronically diabetic myocardium is more susceptible to ischaemic injury. Increased resistance to ischaemic damage was also observed in hearts from both obese and lean type 2 diabetic rats [[13],[31]] and rabbits [[11]].

Interestingly, the degree of functional recovery during reperfusion after 35 min of regional ischaemia did not reflect the reduction in infarct size observed in the obese groups (see Figure 1) and similar values were obtained in the three NPC groups (Table 3). The reasons for these findings are not clear yet. The similar functional recoveries observed in these three NPC groups may be due to the presence of stunning during early reperfusion, which may mask any possible effects of the diet [[41]]. The activation patterns of ERKp44/p42 and PKB/Akt during early reperfusion of hearts from the non-preconditioned NDC, DIO and HFD groups did not reveal any correlation between infarct size reduction in the diet groups and activation of the RISK pathway In fact, the lowest activation of ERKp44/p42 and PKB/Akt was seen in the HFD NPC hearts, while their infarct sizes were significantly smaller than those of the NDC hearts (Figures 1 and 5A,B).

The finding of an increased capacity to tolerate ischaemia after 16 weeks of the DIO diet is in contrast to previous findings in rats on a similar diet [[18],[20]-[22]]. However, prolonging the period of administration of the DIO diet from 16 to 32 weeks, also paradoxically improved the tolerance of the hearts to I/R damage [[27]]. The latter group also reported an increased activation in baseline PKB/Akt (evaluation of activation during reperfusion was not done). In contrast to the DIO study, Du Toit and coworkers reported a very significant reduction in ischaemic tolerance in hearts from rats exposed to a HFD for 32 weeks [[19]]. This increase in infarct size was associated with a reduction in basal PKB/Akt and GSK-3β phosphorylation. However, in the present study baseline expression and activation of ERKp44/p42 and PKB/Akt were similar in the three groups studied [Lochner A, Genade S, Unpublished observations]. The marked difference between our finding of increased tolerance to ischaemia in hearts from HFD rats and the findings of Wensley [[19]] may be due to the period of diet administration (16 vs 32 weeks), differences in diet fat and carbohydrate contents (the Wensley diet contained higher fat and carbohydrate contents) as well as differences in activation of the RISK pathway.

The mechanism whereby moderate obesity and insulin resistance induces a preconditioned state of the heart is still unsure. Possibilities include formation of new collaterals, as was reported in rats [[31]] or rabbits [[11]], a reduction in the production in glycolytic metabolites [[32]] or a larger response of the KATP channel, as was shown in diabetic animals [[11]]. However, the rats used in the present study were insulin-resistant, but not diabetic. The high circulating insulin concentrations of the diet groups (Table 2) may contribute to a permanent preconditioned state. Insulin is known to have a preconditioning effect [[35]]. Another possibility is involvement of the renin-angiotensin system as was demonstrated in early overnutrition in rats [[25]]. This is supported by the finding that angiotensin-converting enzyme inhibition (ACE-I) improved pre-ischaemic left ventricular contractility and restored delayed preconditioning in ob/ob and combined leptin and LDL receptor-deficient mice [[49],[50]]. However, role of these factors in the DIO and HFD hearts have to be investigated.

### Effect of preconditioning

Reports on the capacity of IPC to protect hearts from diabetic animals are contradictory, while its effect on hyperphagia-induced obesity has not yet been studied. In addition to IPC, we also evaluated the effects of BPC by using a β2 adrenergic receptor agonist, formoterol, which has been shown to be very effective in eliciting cardioprotection [[42]]. Interestingly, in contrast to hearts from the NDC group, both IPC and BPC had no further reducing effects on the infarct sizes of DIO and HFD hearts. This could be due to the fact that these hearts were already maximally protected by their respective diets. However, functional recovery did improve, particularly in the DIO hearts, after both types of preconditioning, when compared with its respective non-preconditioned group. This was also reflected in the number of hearts that were able to produce aortic flow during reperfusion (see Table 3).

Comparison of the effects of IPC and BPC with NPC on activation of the RISK pathway during reperfusion in the three groups are shown in Figures 8, 9 and 10. A clear-cut pattern between a reduction in infarct size as endpoint and ERK and PKB/Akt activation did not emerge: The absence of activation of the RISK pathway by IPC in hearts from the NDC group may be due to the fact that these hearts were preconditioned by a one cycle (1×5 min I/5 min reperfusion) only, since most studies on activation of this pathway were done after multiple cycles of preconditioning [[34],[35]]. Interestingly, in all three groups, BPC caused ERKp44/p42 phosphorylation when compared with NPC hearts (see Figures 8, 9 and 10), but only in the case of NDC was this associated with a reduction in infarct size. No increased activation of PKB/Akt was observed in any of the preconditioned groups, when compared with the corresponding NPC hearts. Thus activation of the prosurvival kinases during reperfusion, characteristic of many interventions associated with cardioprotection [[34],[35]], does not occur in the cardioprotection associated with obesity. Involvement of the mitochondrial permeability transition pore in this scenario also needs to be determined.

Other factors, for example, hypercholesterolemia, hyperglycemia or hypertension [[28]], are unlikely to be the causes for the failure to precondition the heart, since the diet groups were not hypercholesterolemic, hypertensive (data not shown) or hyperglycemic (Table 2). As mentioned previously, the role of the RAS system in this regard needs to be evaluated.

## Conclusions

The results obtained in this study demonstrated that diet-induced obesity for a period of 16 weeks was sufficient to elicit increased tolerance to ischaemia. Since a clear correlation between infarct size reduction, functional recovery and activation of the RISK pathway did not emerge, further studies are required to elucidate the scientific basis for the “obesity paradox”. Indeed recent studies showed that a high sucrose diet induced profound changes in myocardial glucose metabolism and suggested further exploration of mechanisms regulating substrate metabolism in the insulin resistant heart [[46]].

## Abbreviations

NDC: Non-diet controls

DIO: Diet-induced obesity

HFD: High fat diet

NPC: Non-preconditioned

IPC: Ischaemic preconditioning

BPC: Beta-adrenergic preconditioning

## Competing interests

The authors declare that they have no competing interests.

## Authors’ contributions

AL conceived the study, designed the protocol, did the statistical analyses and drafted the manuscript; BH helped to draft the manuscript; RS did the perfusions and Western blotting. All authors read and approved the final manuscript.
